# Predictive risk factors of postoperative urinary incontinence following holmium laser enucleation of the prostate during the initial learning period

**DOI:** 10.1590/S1677-5538.IBJU.2015.0477

**Published:** 2016

**Authors:** Shuichiro Kobayashi, Masataka Yano, Takayuki Nakayama, Satoshi Kitahara

**Affiliations:** 1 Department of Urology, Tama-Nambu Chiiki Hospital, Tokyo, Japan

**Keywords:** Blood Loss, Surgical, Lasers, Learning, Prostatic Hyperplasia, Urinary Incontinence

## Abstract

**Purpose:**

To determine the predictive factors for postoperative urinary incontinence (UI) following holmium laser enucleation of the prostate (HoLEP) during the initial learning period.

**Patients and Methods:**

We evaluated 127 patients with benign prostatic hyperplasia who underwent HoLEP between January 2011 and December 2013. We recorded clinical variables, including blood loss, serum prostate-specific antigen levels, and the presence or absence of UI. Blood loss was estimated as a decline in postoperative hemoglobin levels. The predictive factors for postoperative UI were determined using a multivariable logistic regression analysis.

**Results:**

Postoperative UI occurred in 31 patients (24.4%), but it cured in 29 patients (93.5%) after a mean duration of 12 weeks. Enucleation time >100 min (p=0.043) and blood loss >2.5g/dL (p=0.032) were identified as significant and independent risk factors for postoperative UI.

**Conclusions:**

Longer enucleation time and increased blood loss were independent predictors of postoperative UI in patients who underwent HoLEP during the initial learning period. Surgeons in training should take care to perform speedy enucleation maneuver with hemostasis.

## INTRODUCTION

Transurethral resection of the prostate (TURP) is considered a standard procedure for treating benign prostatic hyperplasia (BPH). In recent years, several laser systems and applications have been applied to the endoscopic surgical treatment of BPH. Holmium laser enucleation of the prostate (HoLEP) is one such type of laser treatment. It was first reported by Gilling et al. (1) and several studies have documented its efficacy and safety (2-4). This minimally invasive technique enables complete prostate resection even in cases with a large prostate volume (5, 6). In addition, it is reported to provide better short-term urinary functional outcomes, shorter duration of Foley catheter use, and shorter hospital stay than TURP (7). HoLEP is expected to become the new gold standard technique for treating BPH. However, difficulties of surgical techniques and high frequency in postoperative urinary incontinence (UI) remain to be significant problems for unexperienced urologists.

HoLEP required a certain surgical learning curve. Some studies have reported that urologists need a threshold of at least 20-50 cases to gain acceptable efficacy (8, 9). Although HoLEP is one of the established surgical procedures, not a few urologists hesitate to introduce HoLEP techniques. A multicenter prospective study showed that almost half of centers which introduced Ho-LEP chose to terminate the operation due to longer operative time and difficulty of enucleation of the prostatic lobe (10).

Postoperative UI is mainly classified as either stress or urge UI. These UI occurred after BPH surgery by several factors, the etiology still remains to be elucidated. Transient stress UI after HoLEP is similar to that observed after TURP according to a meta-analysis comparing HoLEP and TURP (7). On the other hand, short-term urge UI after HoLEP is more frequent than those of TURP (4). Almost all UI cases improved after a few weeks, but persistent UI occurs in 1%–2% patients (2, 4, 11).

There is little available data on the relationship between perioperative variables including surgical parameters and UI following HoLEP (11, 12). To disseminate and continue with the technique of HoLEP, it is important to determine the cause of postoperative UI. Thus, in the present study, we evaluated the clinical characteristics that can significantly predict postoperative UI following HoLEP, particularly during the initial learning period.

## PATIENTS AND METHODS

The present study was approved by the institutional review board of our hospital. A total of 146 initial patients who underwent HoLEP between January 2011 and December 2013 were identified using a prospectively collected database. The HoLEP procedure was performed either of two surgeons who were skilled in endourological procedures. We excluded 12 patients of conversion to TURP due to capsular perforation or uncontrolled bleeding. We also excluded patients who were unable to answer the questionnaires regarding their UI because of dementia (n=2) and those who presented with UI preoperatively (n=5). Thus, a total of 127 patients were eligible for the present study.

Subjective symptoms of the International Prostate Symptom Score (IPSS) and quality of life (QoL) scores, objective parameters of uroflowmetry, and post-void residual urine (PVR) were measured pre- and post-operatively. The total prostate volume was determined by transrectal ultrasonography. Transperineal prostate biopsy was performed to exclude prostate cancer when serum prostate-specific antigen (PSA) >4.0ng/mL. IPSS, QoL scores, uroflowmetry, and PVR were determined at 3 months after HoLEP and were compared with preoperative data.

Our HoLEP technique was based on the procedure described in detail by Gilling et al. (1). We used a 100W holmium: YAG laser source (VersaPulse PowerSuite, Lumenis, Yokneam, Israel), a 550μm laser fiber (SlimLine, Lumenis, Yokneam, Israel), and a 26Fr continuous-flow resectoscope (Karl Storz, Tuttlingen, Germany). We placed the patients generally under spinal anesthesia and dissected and enucleated the median, left, and right lobes of the prostate one by one in a retrograde fashion. We performed morcellation of the 3 enucleated lobes using a tissue morcellator (VersaCut, Lumenis, Yokneam, Israel). After the procedure, a 20Fr Foley catheter was placed and the urinary bladder was continuously irrigated until the next day. The patient was typically discharged after the removal of the Foley catheter on postoperative day 2.

We evaluated preoperative variables in all patients, including age, body mass index (BMI), diabetes mellitus, serum PSA levels, use of medication including antiplatelet agents, history of acute urinary retention, and total prostate volume (Table-1). In patients receiving antiplatelet therapy, these agents were usually terminated 7–10 days before HoLEP, and then resumed 1–2 days after surgery. We also noted surgery-related variables, including operative time, enucleation time, morcellation time, total energy, occurrence of bladder injury, enucleated prostate volume and blood loss (Table-1). The difference in hemoglobin levels between before surgery and on postoperative day 1 was used to estimate blood loss. The postoperative PSA levels were obtained at the 3-month follow-up visit, and the percentage reduction in PSA levels was calculated using serum PSA levels obtained before and after surgery. We observed the patients for postoperative complications such as acute urinary retention, blood transfusion, UI, urethral stricture, and urinary tract infection for 90 days after HoLEP.

**Table 1:** Baseline characteristics of 127 patients.

		Mean ± SD or N
**Preoperative variables**		
	Age, year	72 ±6
	BMI, kg/m^2^	23.2 ± 2.9
**Diabetes mellitus**		
	Yes	20
	No	107
PSA, ng/mL		7.0 ±6.3
**Antiplatelet agents**		
	Yes	9
	No	118
**History of acute urinary retention**		
	Yes	41
	No	86
Total prostate volume, mL		69 ±33
**Surgery-related variables**		
	Operative time, min	127 ±50
	Enucleation time, min	93 ±38
Morcellation time, min		14 ± 12
Total energy, KJ		133.0 ±47.8
**Bladder injury**		
	Yes	6
	No	121
Enucleated prostate volume, g		40 ±27
Blood loss, g/dL		1.4 ± 1.2
**Postoperative variables**		
	PSA reduction, %	82.8±14.0

**BMI** = body mass index; **PSA** = prostate-specific antigen; **SD** = standard deviation.

All patients were asked about the presence or absence of UI at every outpatient visit before and after HoLEP. Stress UI was evaluated on the basis of medical interview. Urge UI was assessed by using the overactive bladder symptom score, the symptom assessment questionnaire of overactive bladder: daytime frequency, nocturia, urgency, and urge incontinence (13). On the basis of the responses to questions, we excluded patients with preoperative UI, and we collected information regarding the type of UI (stress UI, urge UI, or mixed UI) and the total number of pads used per day. Continence was defined as complete dryness or 1 pad used prophylactically per day.

All variables were analyzed for statistically significant differences using the Mann-Whitney U test for continuous variables and the chi-square and Fisher exact tests for categorical variables. To identify the risk factors for the incidence of postoperative UI following HoLEP, a logistic regression analysis was used, and odds ratios (ORs) and 95% confidence intervals (CI) were determined. P values <0.05 on a univariate analysis were included in a multivariate logistic model. The statistical analyses were performed using JMP version 9.0 (SAS Institute Inc., Cary, NC, USA). All p values <0.05 were considered significant.

## RESULTS

Table-1 illustrates the clinical characteristics of all 127 patients. The mean blood loss was 1.4g/dL. Nine patients continued antiplatelet therapy during surgery. Mean IPSS (p<0.0001), mean QoL scores (p<0.0001), and mean PVR rate (p<0.0001) had significantly decreased from baseline, respectively. Similarly, mean Qmax rate significantly increased (p<0.0001). We evaluated the correlations between blood loss and clinical variables. When patients were divided into groups on the basis of a blood loss >2.5g/dL (n=18) and other parameters (n=109), larger total prostate volume (p<0.0001), longer operative time (p=0.0098), longer morcellation time (p=0.0002), and larger enucleated prostate volume (p<0.0001) were significantly correlated with a blood loss >2.5g/dL. In contrast, blood loss was not correlated with the status of antiplatelet drug use and the percentage reduction in PSA.

The mean follow-up was 13 months (range, 3–53 months). A total of 31 patients (24.4%) developed postoperative UI as follows: 17 with stress UI, 9 with urge UI, and the remaining 5 with mixed UI. After pelvic floor exercises and/or anticholinergic drugs introduced in these patients, UI disappeared in 29/31 (93.5%) patients at a mean duration of 12 weeks (range, 2–28 weeks). Two patients with mixed UI had persistent incontinence until the last follow-up visit (>2 year).

Overall, perioperative complications occurred in 22 (17.3%) patients. Superficial bladder mucosal injury during morcellation, urethral stricture, febrile urinary tract infection, and postoperative acute urinary retention developed in 6, 7, 7, and 1 patient, respectively. One patient who had a prostate volume of 202mL required a blood transfusion. After HoLEP, his hemoglobin level decreased from 13.0g/dL to 8.3g/dL on postoperative day 1. When his hemoglobin level was 7.4g/dL on postoperative day 2, we transfused 2 units of packed red blood cells without any adverse events.

Further, we assessed the clinical parameters that were significantly associated with postoperative UI. Univariate analysis revealed that enucleation time >100 min, enucleated prostate volume >50g, and increased blood loss >2.5g/dL were significantly associated with postoperative UI. We found no significant difference between both surgeons and incidence of postoperative UI (data not shown). On multivariable logistic regression analysis, enucleation time >100 min (OR, 2.54; 95% CI, 1.03–6.30; p=0.043) and increased blood loss >2.5g/dL (OR, 3.62; 95% CI, 1.12–11.99; p=0.032) were identified as independent and significant predictors of postoperative UI (Table-2). In sub-analysis of associations between two types of UI and clinical parameters, univariate analysis revealed that enucleated prostate volume >50g (p=0.030), and increased blood loss >2.5g/dL (p=0.017) were associated with stress UI, and increased blood loss >2.5g/dL was only identified as a significant parameter associated with urge UI (p=0.030).

**Table 2:** Changes in clinical parameters after HoLEP.

	Preoperative	Postoperative	
	Mean ± SD	Mean ± SD	p
IPSS	18.5 ±8.3	5.9 ± 5.8	<0.0001
QoL	4.7 ±1.3	1.6 ±1.5	<0.0001
Qmax, mL/sec	9.3 ±4.3	21.8 ±11.2	<0.0001
PVR, mL	144.2 ±266.1	17.9 ±29.1	<0.0001

**IPSS** = International Prostate Symptom Score; **PVR** = post-void residual urine; **Qmax** = maximal flow rate; **QoL** = quality of life; **SD** = standard deviation

## COMMENTS

There is still limited data regarding the predictive factors for stress UI in patients following HoLEP. Detrusor dysfunction, sphincter incompetence, and mixed incontinence have been considered as the main etiological factors for stress UI after prostatectomy. Long-term urinary bladder outflow obstruction leads to detrusor instability, and any damage to the urethral sphincter during surgery results in sphincter incompetence (14). Elmansy et al. (12) demonstrated that total prostate volume, operative time, and percentage reduction in PSA levels were significantly associated with stress UI after HoLEP. One possible explanation for the incidence of stress UI is that large prostate volumes are associated with longer operative times. This is associated with longer durations during which the sheath is manipulated across the urethral sphincter, which may cause sphincter incompetence (12). Another possible explanation is that more complete prostate tissue removal creates a large prostatic fossa and causes short-term urine trapping and leakage during stress maneuvers (15). A greater percentage reduction in PSA levels may be a surrogate marker of less residual adenoma following BPH surgery, which could lead to the incidence of stress UI seen after HoLEP.

There are several hypotheses regarding the mechanism of postoperative urge UI. Patients with preoperative terminal detrusor overactivity are more likely to develop high overactive bladder symptom scores and persistent urge UI after TURP (16). On the other hand, detrusor overactivity is not associated with urge UI following HoLEP, and the presence of bladder injury during morcellation was the only predictive factor for urge UI (11). Although it is important to provide hemostasis for a clear endoscopic view for avoiding bladder mucosal injury during morcellation, we did not identify a significant difference between intraoperative bleeding and bladder mucosal injury in our cohort.

Surgical technique of HoLEP during the learning period is one major predictor of postoperative UI (Table-3). Although HoLEP has become increasingly utilized for the treatment of BPH, the technical challenge of HoLEP is greater than those of TURP and requires longer training periods (8). The occurrence of postoperative UI after Ho- LEP was 4.9–12.5% with even expert surgeon who operated more than 900 cases (2, 12). The possible technical causes of developing stress UI during the learning phase are thought to be violate operative planes, enucleation too deep, and over-dissection at the level of apex (17). In particular, appropriate apical dissection is considered an important point for avoiding urinary sphincteric injury during HoLEP procedure (18). In the present study, surgery-related variables such as enucleation time and blood loss were associated with postoperative UI, whereas all preoperative variables were not. This result indicated technical improvement possibly contributes to reduce postoperative UI. Indeed, Endo et al. (19) revealed that the procedure of anteroposterior dissection (a surgical procedure where adenoma is dissected antegradely) could decrease postoperative stress UI. Further studies are warranted to assess the association between surgical technique and postoperative UI.

**Table 3:** Univariate and multivariate logistic regression analysis for predicting postoperative urinary incontinence.

	Univariate analysis		Multivariate analysis	
Variables	Odds ratio (95% CI)	p value	Odds ratio (95% CI)	p value
Age, y (>75 vs ≤75)	1.16 (0.47-2.73)	0.74		
BMI, kg/m^2^(>25vs≤25)	0.99 (0.39-2.37)	0.99		
Diabetes mellitus (yes vs no)	0.74 (0.20-2.23)	0.61		
PSA, ng/ml (>4 vs ≤4)	1.06 (0.46-2.53)	0.89		
Antiplatelet agents (yes vs no)	0.88 (0.13-3.87)	0.87		
History of acute urinary retention (yes vs no)	1.00 (0.41-2.34)	0.99		
Total prostate volume, ml (>80 vs ≤80)	2.12 (0.88-5.04)	0.092		
Operative time, min (>160 vs ≤160)	1.70 (0.68-4.12)	0.25		
Enucleation time, min (>100 vs ≤100)	2.81 (1.21-6.58)	0.017	2.54(1.03-6.30)	0.043
Morcellation time, min (>20 vs ≤20)	1.11 (0.40-2.85)	0.84		
Total energy, KJ (>170 vs ≤170)	1.92 (0.73-4.86)	0.18		
Bladder injury (yes vs no)	0.61 (0.03-3.96)	0.64		
Enucleated prostate volume, g (>50 vs ≤50)	3.56 (1.51-8.51)	0.0039	2.13(0.78-5.61)	0.14
Blood loss, g/dl (>2.5 vs ≤2.5)	5.24 (1.85-15.34)	0.0019	3.62(1.12-11.99)	0.032
PSA reduction, %(>85 vs ≤85)	1.89 (0.79-4.80)	0.15		

**BMI** = body mass index; **CI** = confidence interval; **PSA** = prostate-specific antigen

Our results generate the hypothesis that blood loss may serve as a predictive factor for the incidence of postoperative UI. Although surgical techniques for controlling bleeding in prostatectomy procedures have been established, blood transfusion is still required in some patients. Martin et al. (20) reported that 8 of 130 patients (6.7%) required postoperative blood transfusions. Intraoperative poor visibility attributed to bleeding increased the risk of misfiring the laser or using excessive compression with the beak of the resectoscope sheath on the external urinary sphincter. This may have resulted in sphincter damage, which lead to stress UI. Moreover, prostatic capsule was exposed to excessive laser energy for stop bleeding during surgical procedure, that may also be attributed to development of urge UI. Thus, we supposed that meticulous hemostasis, without blind laser application, and careful blunt dissection can achieve less bleeding and lead to a decrease in the incidence of postoperative UI.

The present study had several limitations. First, it enrolled a relatively small number of patients; therefore, further evaluation and validation of our study findings is required. Second, HoLEP was not performed by a single surgeon. Although the incidence of postoperative UI was not significantly different between both surgeons, surgical technique potentially influenced functional outcomes. Third, the objective assessment of the presence of detrusor overactivity or sphincter disorders was limited because this study was retrospective in nature, and urodynamic tests other than uroflowmetry were not performed routinely.

## CONCLUSIONS

Our data demonstrated that HoLEP could gain good functional outcomes regardless of duration of the initial learning period. Longer enucleation time and increased blood loss were significantly associated with postoperative UI following HoLEP. Although postoperative UI is usually transient and resolves within a few months, prolonged, severe UI occurred in some patients. Thus, surgeons in training should take care to achieve speedy enucleation with meticulous hemostasis during surgical procedures, and technical improvement of surgery might provide decrease of the incidence of UI.

## Abbreviations

BMI: = body mass index
BPH: = benign prostatic hyperplasia
CI: = confidence intervals
HoLEP: = Holmium laser enucleation of the prostate
IPSS: = International Prostate Symptom Score
ORs: = odds ratios
PSA: = prostate-specific antigen
PVR: = post-void residual urine (PVR)
QoL: = quality of life
TURP: = Transurethral resection of the prostate
UI: = urinary incontinence
